# SwitchClass distinguishes baseline- and perturbation-aligned features across intermediate molecular states

**DOI:** 10.1016/j.crmeth.2026.101547

**Published:** 2026-08-12

**Authors:** Di Xiao, Siqu Long, Pengyi Yang

**Affiliations:** 1 Computational Systems Biology Unit, Children’s Medical Research Institute, Faculty of Medicine and Health, The University of Sydney, Westmead, NSW 2145, Australia; 2 Sydney Precision Data Science Centre, The University of Sydney, Sydney, NSW 2006, Australia; 3 School of Mathematics and Statistics, Faculty of Science, The University of Sydney, Sydney, NSW 2006, Australia; 4 Charles Perkins Centre, The University of Sydney, Sydney, NSW 2006, Australia

**Keywords:** label-switch classification, directional feature importance, intermediate molecular states, multi-state omics, perturbation response

## Abstract

Biological systems often exhibit intermediate molecular states under perturbation, showing changes that align with baseline condition or perturbed state. Capturing these complex patterns is critical for understanding molecular resilience and maladaptive persistence. We introduce SwitchClass, a label-switch classification framework that distinguishes features whose intermediate-state profiles align with baseline or perturbed condition. By training a dual classifier with inverted labels, SwitchClass computes a directional importance score (*δ*), which quantifies each feature’s alignment across biological states. Applied to colorectal cancer proteomics, SwitchClass reveals proteins that normalize after therapy and those remaining dysregulated, uncovering partial molecular recovery. In dietary perturbation and reversal phosphoproteomics, it uncovers the phosphorylation sites linked to incomplete restoration of insulin signaling. In single-cell transcriptomes from COVID-19 patients with varying severities, it identifies cell-type-specific transcripts marking resolution or persistence of inflammatory activity. Together, these analyses demonstrate SwitchClass as an interpretable framework for mapping directional molecular changes in systems with intermediate states.

## Highlights

• SwitchClass scores directional alignment in intermediate molecular states
• Label-switch classifiers identify baseline- or perturbation-aligned features
• Simulations support robust detection of directional feature classes
• Applications reveal recovered, persistent, and severity-linked molecular programs

## Motivation

Intermediate molecular states are common in studies of treatment response, dietary reversal, and disease severity, but standard differential analyses are mainly designed to compare pairs of conditions. As a result, they do not directly identify which features in an intermediate state resemble the baseline condition and which align with the perturbations. We developed SwitchClass to address this need by converting a three-state problem into complementary label-switch classifiers and contrasting feature-importance scores. This yields an interpretable directional score for prioritizing baseline-aligned and perturbation-aligned molecular features across omics datasets.

## Graphical abstract

**Figure:**
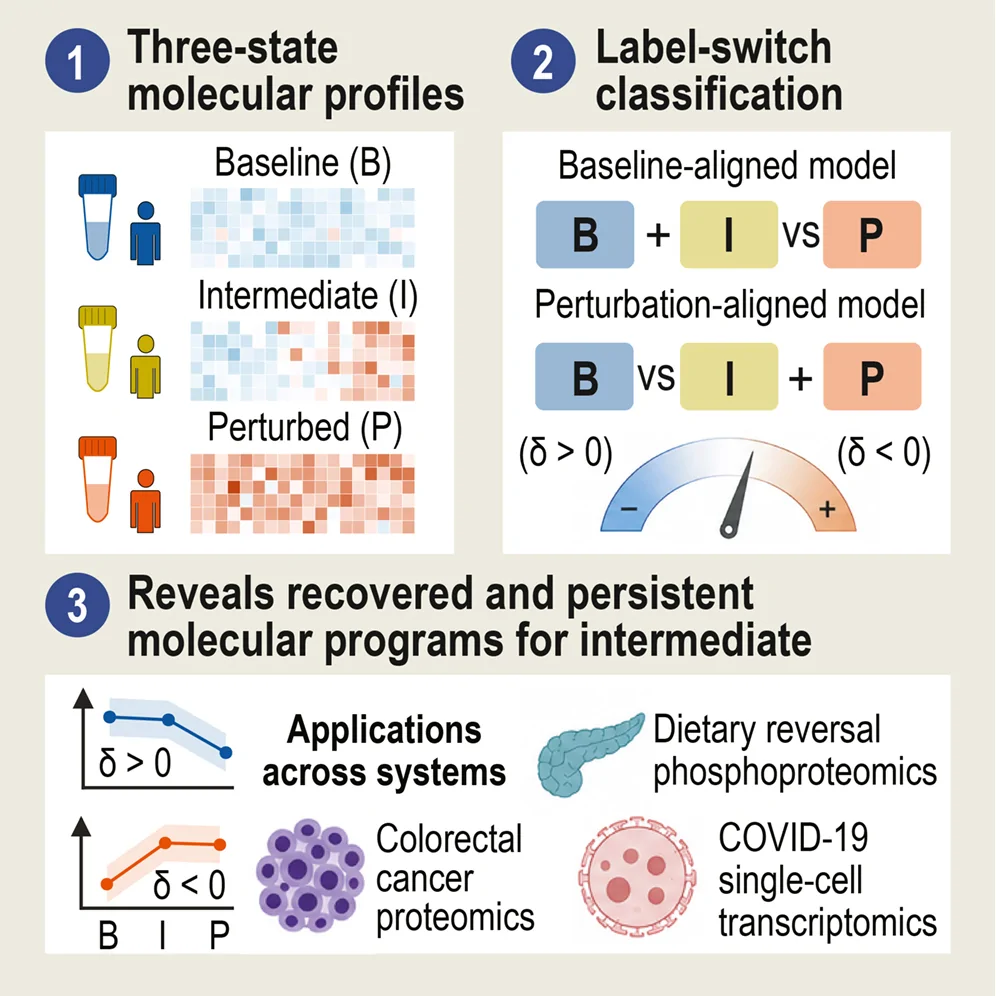

## Teaser

Xiao et al. present SwitchClass, a label-switch classification framework that scores whether molecular features in intermediate states align with baseline or perturbation. Across proteomic, phosphoproteomic, and single-cell transcriptomic datasets, SwitchClass reveals recovered and persistent molecular programs after cancer treatment, dietary reversal, and COVID-19.

## Introduction

Biological systems often respond to perturbations through diverse molecular changes that can restore equilibrium or reinforce altered functional states.^1^ At the molecular level, such perturbations remodel signaling and transcriptional networks, producing patterns in which some features remain close to a baseline profile and others diverge toward perturbed states. In cancers, large-scale proteomic profiling has shown that therapeutic intervention induces heterogeneous molecular responses, with some proteins returning toward baseline and others remaining dysregulated despite treatment.^2^ In metabolic systems, phosphoproteomic analyses under dietary perturbation and reversal have revealed phosphorylation sites that behave consistently with insulin-sensitive signaling and others that retain insulin-resistant characteristics.^3^ In immune contexts, single-cell transcriptomic studies of COVID-19 have highlighted substantial variation in the inflammatory and interferon activity across healthy, mild, and severe cases.^4^ Across these diverse settings, an intermediate molecular state often emerges, whether representing partial recovery, incomplete reversal, or a milder perturbation, in which subsets of molecular features exhibit alignment with either the baseline condition or the perturbed condition. Such patterns reveal the structure of molecular resilience and divergence, yet remain difficult to quantify systematically.

Existing analytical frameworks provide limited tools for capturing these directional relationships. Widely used methods in transcriptomics and proteomics, such as limma-voom^5^ and edgeR,^6^ have been designed for pairwise comparisons and excel at detecting features that differ between two states. Their single-cell extensions, including NEBULA^7^ and muscat,^8^ similarly focus on pairwise differences. Although powerful for statistical testing, these approaches do not quantify how features in an intermediate condition align, whether they behave more like the baseline state or more like the perturbed one. Trajectory-based methods such as tradeSeq^9^ and Monocle^10^ infer gene dynamics along continuous developmental or pseudotemporal paths, but they assume ordered progression and are not well suited to cross-sectional multi-condition datasets where intermediate states are not defined by temporal ordering. Classification-based models and feature-selection methods^11^ quantify the relevance of features under a given labeling, but standard formulations cannot reveal directional changes of feature importance in an intermediate state. Consequently, standard differential analysis and feature-selection strategies offer limited insight into the directional behavior of molecular features across multiple states, leaving the organization of intermediate conditions largely unexplored.

To address this gap, we developed SwitchClass, a label-switch classification framework for quantifying the directional molecular alignment across three-state systems. SwitchClass builds on the concept that, given two bookend conditions (e.g., baseline and perturbed) and an intermediate state, subsets of molecular features may align preferentially with one end. The framework constructs two complementary binary label assignments: (1) a baseline-aligned labeling, in which intermediate samples are grouped with the baseline and classified against the perturbed state, and (2) a perturbation-aligned labeling, in which intermediate samples are grouped with the perturbed state and classified against the baseline (Figure 1A). A classifier is trained under each labeling on the same feature set, and a directional importance score (*δ*) is computed as the difference between the corresponding feature-importance vectors. The sign of *δ* indicates directional alignment, where a positive sign indicates baseline-aligned features and a negative sign indicates perturbation-aligned ones, and |*δ*| reflects the strength of this alignment. In this study, we instantiate SwitchClass by using random forest classifiers with bootstrap aggregation to stabilize feature-importance estimates, although other learning algorithms may be used, provided they yield feature-importance measures in the label-switch framework.

**Figure 1:**
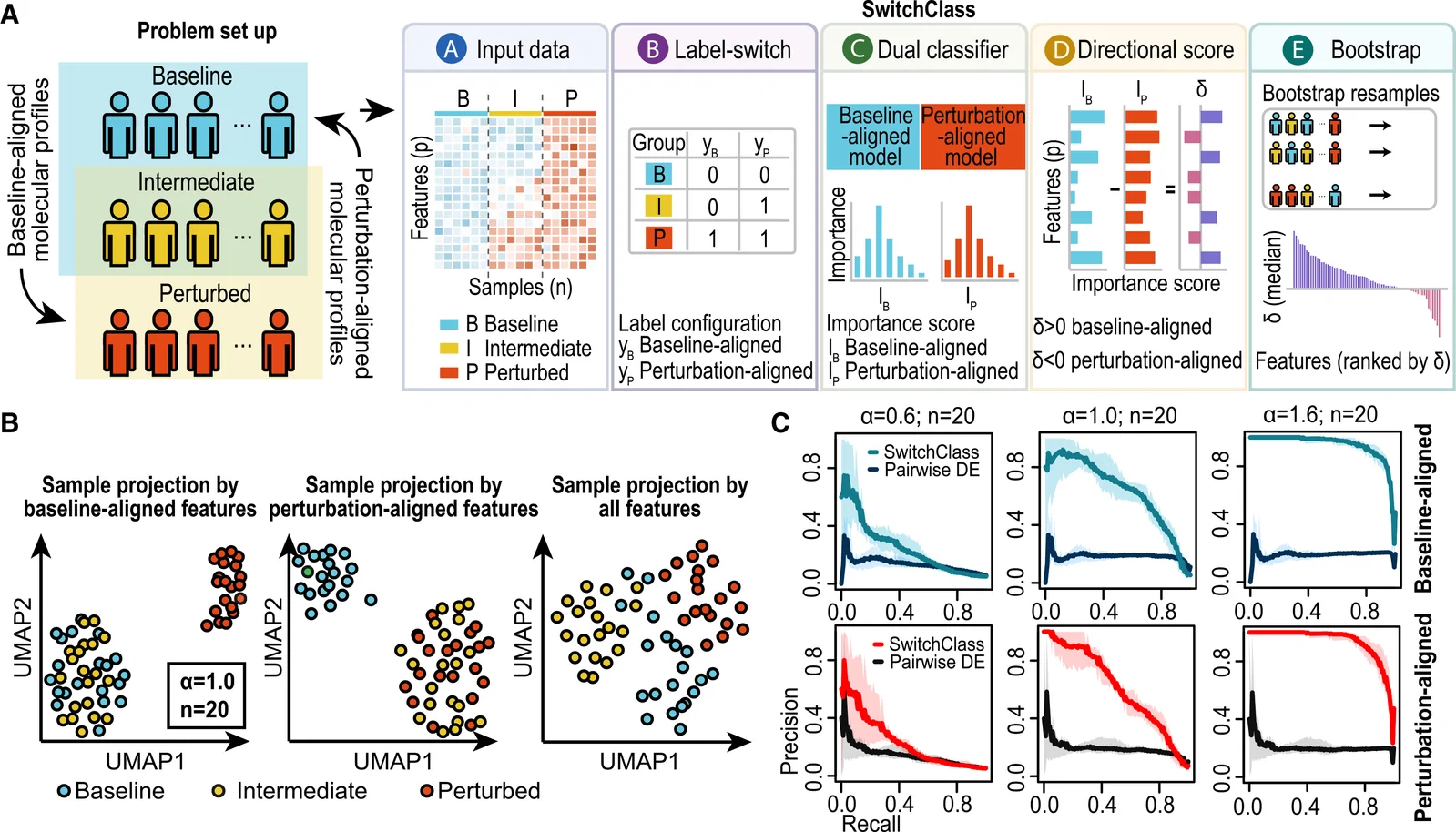
Conceptual overview and benchmarking of SwitchClass (A) Schematic of the problem setup and SwitchClass workflow. SwitchClass models molecular adaptation across three states (baseline, intermediate/recovery, and perturbed) by training two complementary label assignments: a baseline-aligned assignment (*y*_*B*_), in which baseline and intermediate samples are grouped against perturbed samples, and a perturbation-aligned assignment (*y*_*P*_), in which baseline samples are grouped against the intermediate and perturbed samples. The corresponding feature-importance vectors (*I*_*B*_ and *I*_*P*_) are contrasted to compute the directional importance score *δ* = *I*_*B*_-*I*_*P*_, where positive values indicate baseline-aligned features, and negative values indicate perturbation-aligned features. (B) UMAP of the simulated samples using baseline-aligned, perturbation-aligned, and all features (*α* = 1.0; *n* = 20). (C) Precision-recall performance of SwitchClass in detecting baseline-aligned and perturbation-aligned features compared with pairwise differential expression (DE) across varying effect sizes *α* = 0.6, 1.0, 1.6 and a sample size of *n* = 20 for each state. Shaded areas denote 95% confidence intervals derived from bootstrap resampling across simulation replicates.

Through simulations, we show that SwitchClass robustly detects directional signals across varying effect sizes and sample sizes. When applied to mass spectrometry (MS)-based colorectal cancer proteomics spanning healthy controls and patient samples collected before and after treatment, SwitchClass reveals proteins whose abundances normalize following therapy and those that remain persistently dysregulated, implicating processes of partial molecular recovery and sustained tumor-associated activity. In mouse phosphoproteomics profiling dietary perturbation and reversal, the method identifies phosphorylation sites that normalize or remain elevated, uncovering signaling nodes linked to insulin resistance and its incomplete resolution. In single-cell transcriptomes spanning healthy individuals and patients with mild and severe COVID-19, SwitchClass identifies cell-type-specific transcripts in mild cases that either remain comparable to those in healthy individuals or are elevated to levels observed in severe disease cases. These patterns delineate distinct transcriptional states across multiple immune cell types that differentiate disease severity in response to COVID-19. Together, these analyses demonstrate the utility of SwitchClass and position it as a practical and interpretable framework for mapping directional molecular divergence across biological systems.

## Results

### Evaluating SwitchClass on simulated datasets

We first evaluated the performance and robustness of SwitchClass by using simulated datasets where the true behavior of each feature was known. Each dataset comprised three groups representing baseline, intermediate, and perturbed states. A subset of 100 features was simulated to exhibit specific behavior, where for baseline-aligned features, the intermediate group resembled the baseline profiles, and for perturbation-aligned features, it resembled the perturbation state profile. Additional features were generated to show either intermediate-specific deviations (200) or no change (700) (Figure S1A). The mean separation between the baseline and perturbed groups (effect size, *α*) and the number of samples per group (*n*) were systematically varied to test the method performance under different signal and sample-size conditions (see STAR Methods).

As expected, sample projections based on baseline-aligned or perturbation-aligned features reproduced the designed relationships among the three groups, in which uniform manifold approximation and projection (UMAP) embeddings derived from baseline-aligned features grouped the baseline and intermediate samples together and separated them from the perturbed group, whereas embeddings based on perturbation-aligned features grouped the intermediate and perturbed samples and separated them from baseline (Figure 1B). In contrast, intermediate-specific features separated the intermediate group, while null features produced no structured separation (Figure S1B).

Across simulation settings, SwitchClass recovered the expected directional behavior of features, yielding *δ* scores that separate across the simulated categories (i.e., baseline-aligned, perturbation-aligned, intermediate-specific, and null; Figure S1C). Specifically, baseline-aligned features showed positive *δ* scores, and perturbation-aligned features showed negative *δ* scores, whereas intermediate-specific and null features were centered near zero. The magnitude of *δ* scaled with both the effect size (*α*) and the sample size (*n*), reflecting improved directional confidence as the contrast between the baseline and perturbed states strengthened. This behavior demonstrates that the feature-importance score generated by SwitchClass faithfully encodes both the direction and strength of the alignment across states.

To benchmark performance, we compared SwitchClass with a pairwise differential expression (DE) approach that tested baseline against the intermediate samples for perturbation-aligned features and intermediate against the perturbed samples for baseline-aligned features, separately, on the above simulated datasets. Because this pairwise strategy evaluates only one boundary at a time and cannot integrate information across all three states, its ability to resolve directional relationships was limited, particularly when intermediate-specific effects were present. In contrast, SwitchClass achieved substantially higher precision-recall performance across all simulation settings, as demonstrated by the higher precision-recall curves (Figure 1C; Figure S1D). Notably, even under weak signal settings (*α* = 0.6), SwitchClass maintained relatively high precision, demonstrating that the label-switch design effectively leverages information across states to enhance directional inference. These simulation experiments demonstrate that SwitchClass provides a reliable and interpretable measure of the directional behavior of features across states, establishing a robust foundation for its application to empirical omic datasets. While the random forest model was used for the above experiments, we also evaluated other feature-ranking approaches, including elastic-net logistic regression and gradient boosting with SHAP values (Figure S1E). Among the evaluated implementations, the random forest model achieved consistently higher directional detection performance across simulation settings and was used as the default approach in SwitchClass and in subsequent analyses.

We next extended the simulation benchmark to more challenging settings. With random label noise (0%–30% relabeled samples), SwitchClass performance decreased gradually but remained above the random baseline across the tested effect sizes, with stronger signals showing greater robustness (Figure S1F). Block-wise feature correlations (*ρ*∈{0, 0.3, 0.6, 0.9}) had little effect on recovery of the baseline-aligned and perturbation-aligned features (Figure S1G). In partial-recovery simulations, where the mean of the intermediate state varied between the baseline and perturbed endpoints, the mean directional score changed smoothly with recovery fraction *β*, moving from positive baseline-aligned scores toward negative perturbation-aligned scores and approaching zero for intermediate profiles (Figure S1H).

We also assessed statistical calibration and sample-level separation. Under label permutation, the pooled signed null distribution of *δ* was centered near zero, whereas the observed *δ* values for true directional features extended beyond this null range (Figure S1I). This permutation-derived null distribution provides an empirical reference for interpreting the directional score magnitude and enables feature-level empirical *p* values and false discovery rate (FDR)-adjusted *q* values to be reported alongside *δ*. Baseline-aligned and perturbation-aligned feature subsets also showed stronger group separation than the all-feature baseline in both the UMAP embedding and the original high-dimensional feature space, as measured by the average silhouette width (Figure S1J).

### SwitchClass reveals recovered and persistent proteomic signatures post-colorectal cancer treatment

We applied SwitchClass to the plasma proteome dataset from a colorectal cancer cohort comprising healthy controls (“baseline”), pre-treatment (“perturbed”), and post-treatment (“intermediate”) samples (Figure 2A).^2^ This dataset captures the molecular profiles of normal individuals, cancer patients, and partial therapeutic recovery. By computing the directional feature-importance scores (*δ* = *I*_*B*_-*I*_*P*_) under dual-label configurations, SwitchClass quantified, for each protein, whether post-treatment abundance aligned toward the healthy or cancer state, classifying features as baseline aligned (*δ* > 0) or perturbation aligned (*δ* < 0) (Figure 2C; Figure S2A). The resulting projections separated samples by the health and treatment status, indicating a coherent directional structure within the plasma proteome (Figure 2B). This separation was supported by average silhouette width analysis, in which baseline-aligned and perturbation-aligned protein subsets showed stronger group separation than the all-protein set in both the UMAP embedding and the original proteomic space (Figure S2C). Directional scores were also highly consistent after stratified downsampling to balanced group sizes (Spearman’s *ρ* = 0.960; Figure S2D).

**Figure 2:**
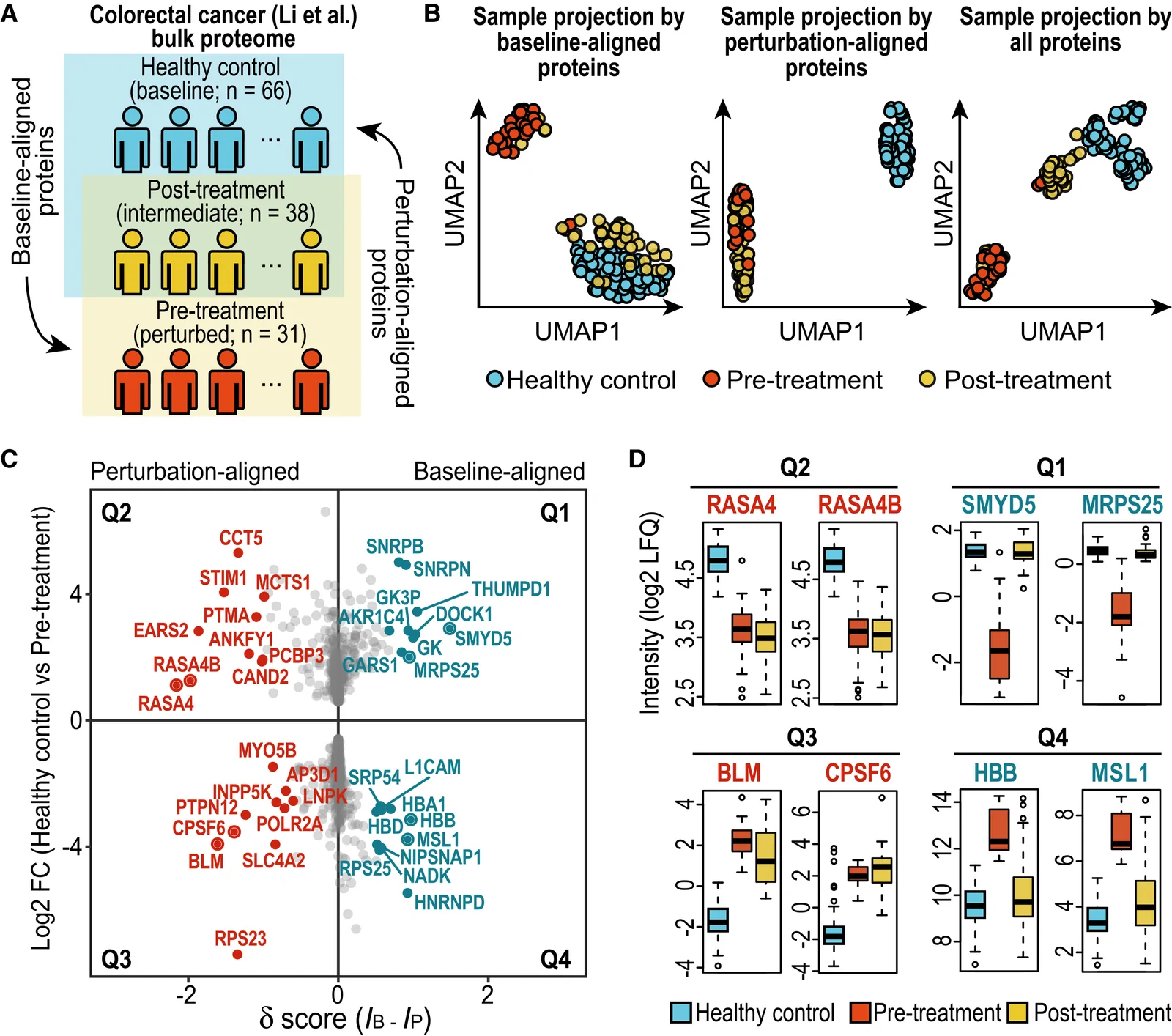
SwitchClass maps directional plasma proteomic changes across colorectal cancer progression and treatment (A) Schematic overview of plasma sample collection from healthy controls (“baseline”), colorectal cancer patients before treatment (“perturbed”), and colorectal cancer patients after treatment (“intermediate”). (B) UMAP visualization of sample projections using baseline-aligned or perturbation-aligned proteins identified by SwitchClass or all proteins. (C) Scatterplot of proteins based on log_2_ fold change (healthy control vs. pre-treatment) and directional importance scores (*δ* = *I*_*B*_-*I*_*P*_) calculated by SwitchClass. Proteins with high |*δ*| in each of the four quadrants are highlighted. (D) Representative proteins from each quadrant illustrating baseline-aligned (Q1/Q4) and perturbation-aligned (Q2/Q3) proteins with distinct recovery or persistence profiles.

Baseline-aligned proteins were enriched in factors involved in mitochondrial function, epigenetic regulation, and tissue homeostasis. Representative baseline-aligned proteins across quadrants Q1 and Q4 include MRPS25 and SMYD5 (Q1), as well as HBB and MSL1 (Q4) (Figures 2C and 2D). MRPS25, a mitochondrial ribosomal subunit required for oxidative phosphorylation (OXPHOS) protein synthesis, returned to healthy control-like levels following treatment. This shift suggests a metabolic transition from glycolysis toward oxidative phosphorylation, a hallmark of recovered energy metabolism in colorectal cancer.^12^ Similarly, SMYD5 functions as a tumor suppressor in colorectal cancer, in part, through its role in chromatin regulation. Its depletion accelerates tumor growth and induces gene expression profiles resembling human colorectal cancer.^13^ Restored SMYD5 expression after treatment indicates re-establishment of epigenetic regulation, particularly heterochromatin integrity. Elevated HBB levels in plasma are associated with heme-related oxidative stress, a common feature in cancer.^14^ Their decline after treatment suggests reduced redox stress and a shift toward metabolic recovery. Finally, the recovery of MSL1, a component of the histone acetyltransferase complex containing lysine acetyltransferase 8 (KAT8/MOF) histone acetylation complex, points to a reversal of chromatin hyperactivation and reinstatement of balanced histone modification activity.^15^ Collectively, these baseline-aligned proteins reflect coordinated recovery of metabolic, epigenetic, and microenvironmental functions following therapy. Pathway enrichment analysis confirmed functional distinctions across quadrants (Figure S2B). Q1 proteins were enriched for chromatin regulation, histone methylation, and proteostasis control, while Q4 proteins were enriched for oxidative stress detoxification, chemical stress response, and amino acid metabolism. These signatures collectively point to systemic rebalancing of metabolic and epigenetic homeostasis as a feature of partial molecular remission.

Perturbation-aligned proteins, by contrast, captured pathways and processes that remained pathologically active in post-treatment tumors. Proteins such as RASA4 and RASA4B (Q2) remained significantly downregulated in both pre- and post-treatment tumors relative to healthy controls, indicating sustained loss of Ras GTPase activity and thus persistent RAS/MAPK pathway activation, thereby promoting malignant progression and drug resistance.^16^ Enrichment analysis identified complement and coagulation cascades, heme-scavenging, and scavenger receptor activity among Q2 proteins, consistent with the ongoing inflammation and disrupted metabolic clearance mechanisms (Figure S2B). Additional perturbation-aligned protein levels were observed for BLM and CPSF6 (Q3) (Figure 2C). BLM, involved in DNA repair, remained high after treatment, suggesting an ongoing DNA damage response or replicative stress in residual tumor cells. Clinically, elevated BLM levels are linked to poorly differentiated colorectal cancer subtypes and shorter relapse-free survival, highlighting its association with aggressive tumor phenotypes.^17^ Likewise, CPSF6, a core regulator of mRNA processing, remained persistently elevated, reflecting continued high transcriptional activity and cell proliferation demands despite treatment. Its sustained expression is linked to poor prognosis and treatment resistance in colorectal cancer, while its knockdown restores drug sensitivity and limits tumor growth.^18^ Q3 proteins were enriched for receptor tyrosine kinase signaling and intracellular stress adaptation pathways, reflecting persistent survival signaling and resistance to molecular resolution.

Together, these results demonstrate that the therapeutic intervention in colorectal cancer elicits a heterogeneous proteomic response. By separating proteins that normalize with treatment from those that remain persistently altered, SwitchClass captures the directional asymmetry of the treatment response and provides an interpretable framework for resolving how recovered and resistant molecular features coexist in treated tumors. This approach delineates a proteomic basis for partial remission, identifying candidate pathways (e.g., RAS/MAPK signaling and DNA repair) that persist after treatment and could be targeted to improve patient outcomes.

### SwitchClass uncovers reverted and persistent signaling nodes in dietary reversal phosphoproteomics

We next applied SwitchClass to a phosphoproteomic dataset profiling the effects of dietary perturbation and reversal in mice (Figure 3A). In this experiment, animals were maintained on a normal chow diet, subjected to high-fat diet (HFD) to induce insulin resistance, and subsequently switched back to chow to enable partial metabolic recovery.^3^ These three groups represent the baseline, perturbed, and intermediate states, providing an experimental model of molecular adaptation and the incomplete restoration of insulin signaling. Phosphoproteome measurements were obtained from chow-fed animals under basal conditions and from all dietary groups following acute insulin stimulation (Figure S3A).

**Figure 3:**
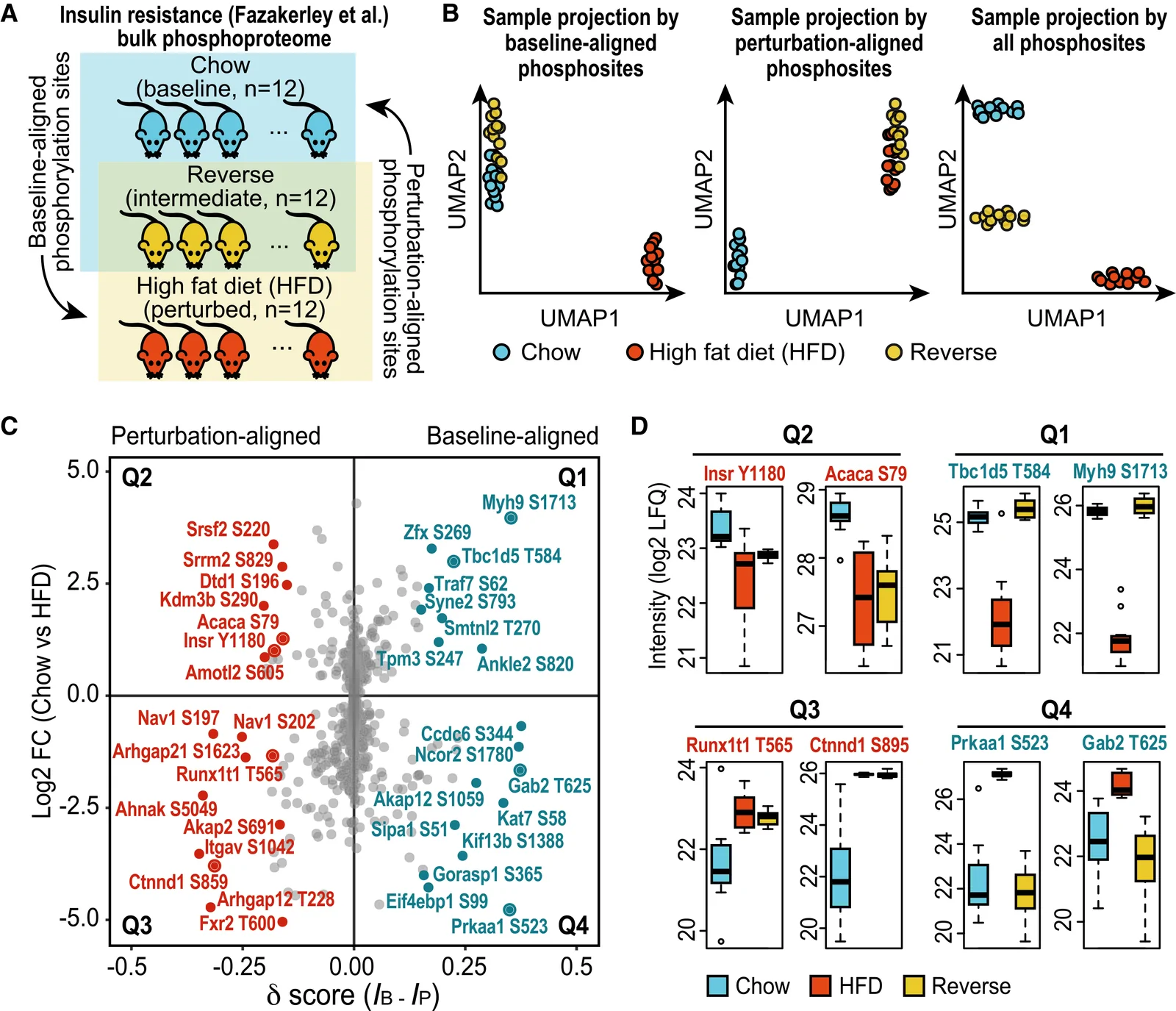
SwitchClass delineates directional phosphorylation states across dietary perturbation and reversal (A) Schematic overview of dissecting phosphoproteomic profiles of mice maintained on a normal chow diet (“baseline”), a high-fat diet (HFD) (“perturbed”), and those subsequently switched back to the chow diet (“intermediate”). (B) UMAP visualization of sample projections using baseline-aligned or perturbation-aligned phosphosites identified by SwitchClass and all phosphosites. (C) Scatterplot of phosphosites by log_2_ fold change (chow diet vs. HFD) and directional importance scores (*δ* = *I*_*B*_-*I*_*P*_) calculated by SwitchClass. Phosphosites with high |*δ*| in each of the four quadrants are highlighted. (D) Representative sites of each of the four quadrants illustrating distinct adaptive behaviors.

Applying SwitchClass to this dataset revealed distinct directional signatures across the phosphoproteome. The method classified phosphosites according to their alignment across dietary states, identifying baseline-aligned and perturbation-aligned features that correctly grouped and separated samples in the UMAP embedding space (Figure 3B). This separation was further supported by the average silhouette width analysis, in which baseline-aligned and perturbation-aligned phosphosite subsets showed stronger group separation than the all-phosphosite set in both the UMAP embedding and the original phosphoproteomic space (Figure S3C). Across the phosphoproteome, baseline-aligned sites were found to be related to canonical insulin-responsive nodes (e.g., AKT, mTOR, and AMPK substrates), consistent with partial restoration of insulin signaling, whereas perturbation-aligned sites comprised persistent insulin-receptor and metabolic phosphorylation events (e.g., INSR and ACACA substrates), highlighting incomplete reversal from HFD-induced signaling (Figure 3C; Figure S3B).

Representative baseline-aligned sites across Q1 and Q4 include Tbc1d5 T584 and Myh9 S1713 (Q1), as well as Prkaa1 S523 and Gab2 T625 (Q4) (Figure 3D). Tbc1d5, a Rab GTPase-activating protein regulating endosomal recycling,^19^ showed normalization, suggesting restored vesicular transport under insulin stimulation. Similarly, Myh9 is related to GLUT4 translocation to the plasma membrane,^20^ and its reversal to baseline suggests recovery of insulin-dependent dynamics for glucose uptake. Dephosphorylation of Prkaa1 S523, an inhibitory site on the AMPK catalytic subunit,^21^ indicates reactivation of cellular energy sensing as insulin sensitivity improves. Finally, normalization of Gab2, a scaffolding adaptor downstream of insulin and cytokine receptors,^22^ reflects reinstatement of balanced receptor-proximal signaling. Collectively, these baseline-aligned sites reflect coordinated restoration of insulin-responsive and metabolic regulatory pathways during dietary reversal.

Conversely, perturbation-aligned phosphorylations were captured in Q2 and Q3 (Figure 3D). Sites such as Insr Y1180 and Acaca S79 (Q2) remained lower in both HFD-fed and reversal animals relative to chow controls, indicating sustained suppression rather than recovery of insulin-receptor and lipogenic signaling.^23^ Additional persistent phosphorylations were observed for Runx1t1 T565 and Ctnnd1 S895 (Q3). Runx1t1, a transcriptional co-regulator promoting adipogenic differentiation,^24^ exhibited elevated phosphorylation in the intermediate state, implying that HFD-induced adipogenic programs persist despite dietary reversal. Ctnnd1, encoding p120-catenin, showed persistent phosphorylation at S895, consistent with ongoing cytoskeletal and junctional remodeling associated with inflammatory signaling.^25^ These Q2–Q3 phosphosites highlight enduring repression of insulin-receptor and metabolic signaling, alongside persistent stress-associated phosphorylation, underscoring that molecular recovery remains incomplete.

Together, these results show that dietary reversal produces a heterogeneous phosphoproteomic response and by separating baseline-aligned features from perturbation-aligned features, SwitchClass captures this directional asymmetry in recovery and provides an interpretable framework for resolving how adaptive and residual signaling coexist during dietary intervention.

### SwitchClass delineates transcriptional states across COVID-19 disease severity

We next applied SwitchClass to a single-cell transcriptomic dataset profiling peripheral blood mononuclear cells from healthy individuals (“baseline”) and patients with mild (“intermediate”) or severe (“perturbed”) COVID-19^4^ (Figure 4A). These three groups represent progressive immune perturbation, from homeostatic to hyperinflammatory states. Single-cell expression matrices were aggregated into pseudo-bulk profiles per cell type and patient (see STAR Methods), and SwitchClass was used to quantify directional transcriptional alignment across severity levels.

**Figure 4:**
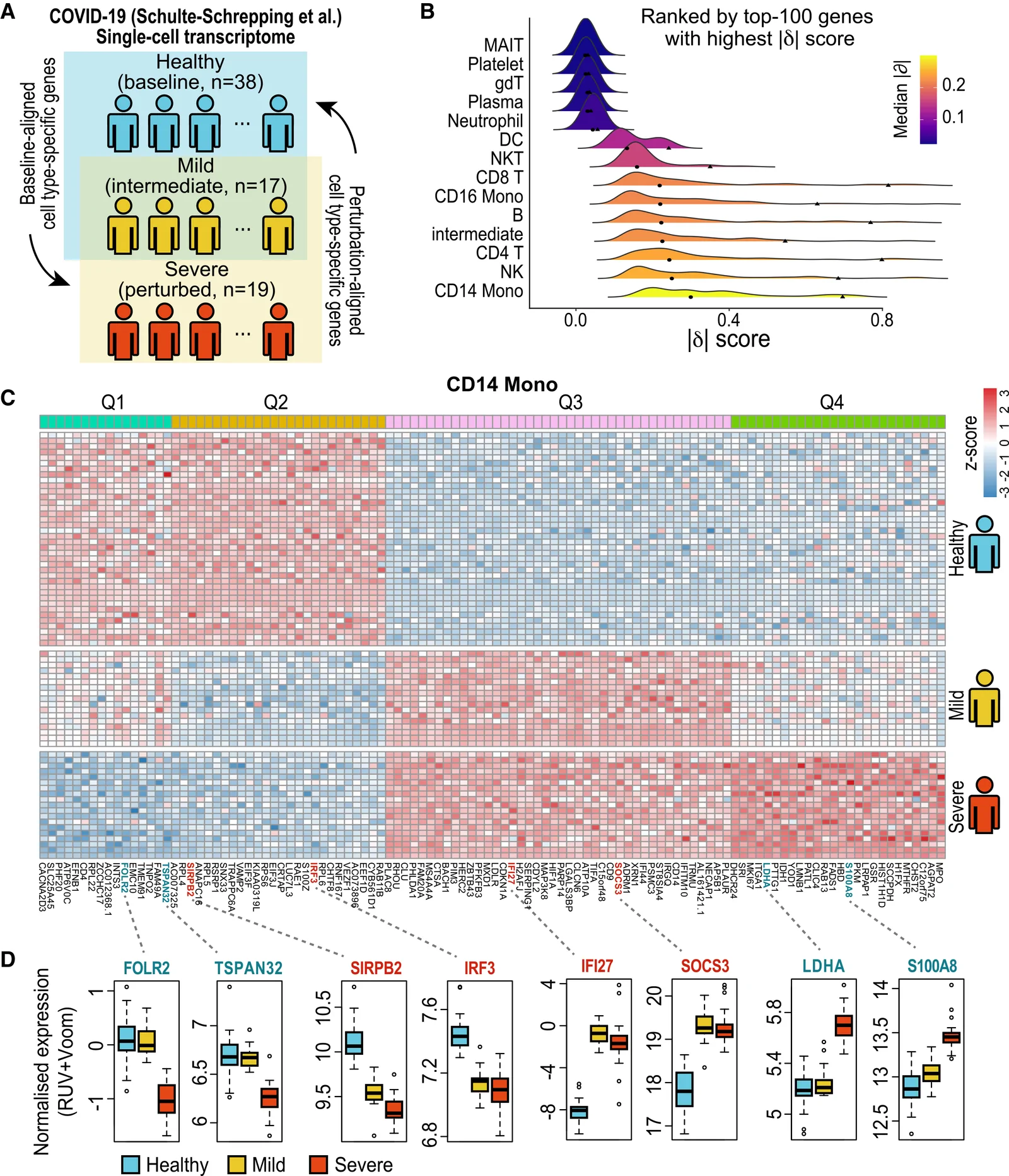
SwitchClass delineates transcriptional states across COVID-19 disease severity (A) Schematic overview of dissecting single-cell transcriptome profiles from healthy individuals (“baseline”) and patients with mild (“intermediate”) or severe (“perturbed”) COVID-19.^4^ (B) Cell-type-specific distributions of directional importance scores (|*δ*|) calculated from the top 100 genes. (C) Heatmap of CD14^+^ monocyte genes identified by SwitchClass across the four quadrants. (D) Representative baseline-aligned (Q1 and Q4) and perturbation-aligned (Q2 and Q3) genes in CD14^+^ monocytes in healthy individuals and patients with mild and severe COVID-19.

Across immune compartments, SwitchClass revealed heterogeneous degrees of molecular divergence (Figure 4B). The magnitude of the directional importance score (|*δ*|) calculated from the top 100 genes varied markedly between cell types, with CD14^+^ monocytes (“CD14 Mono”) and natural killer (NK) cells exhibiting the largest median values, indicating strong directional transcriptional change relative to disease severity. In contrast, mucosal-associated invariant T (MAIT) and platelet cells ranked the lowest, reflecting minimal expression changes and consistent with their restricted transcriptional activity and limited gene expression plasticity in peripheral blood. These patterns are consistent with those in previous studies^4^^,^^26^ and highlight that directional molecular divergence primarily arises within the highly responsive innate compartments, particularly monocytes and NK cells.

We further evaluated the stability of the single-cell SwitchClass results across several analysis choices. Directional scores were largely preserved across different top-gene thresholds, with high Spearman’s correlations and sign concordance between thresholds (Figure S4C). In CD14^+^ monocytes and NK cells, directional scores from the full analysis were also consistent with bootstrap estimates (Spearman’s *ρ* = 0.829 and 0.836, respectively; Figure S4D) and remained highly stable across independent random-seed runs (mean Spearman’s *ρ* = 0.9635 and 0.9602; Figure S4E). Stratified downsampling to balanced group sizes gave similar directional rankings (mean Spearman’s *ρ* = 0.903 and 0.889; Figure S4F), indicating that the main cell-type-specific patterns were not driven by feature-threshold choice, bootstrap sampling, random initialization, or group-size imbalance.

In CD14^+^ monocytes, which displayed the most pronounced directional signatures, SwitchClass stratified genes into four quadrants based on their *δ* values and gene expression patterns (Figure 4C). Representative baseline-aligned transcripts included *FOLR2* and *TSPAN32* (Q1) as well as *LDHA* and *S100A8* (Q4) (Figure 4D). *FOLR2*, encoding a folate receptor enriched in macrophages,^27^ and *TSPAN32*, a tetraspanin implicated in the negative regulation of immune activation and cytokine signaling,^28^ were both reduced in severe disease but maintained in healthy and mild cases, consistent with the suppression of anti-inflammatory and regulatory monocyte programs under hyperinflammatory conditions. Similarly, lower expression of *LDHA*, a key glycolytic enzyme driving inflammatory metabolic reprogramming,^29^ and *S100A8*, a heterodimer constitutively expressed in monocytes and associated with inflammation,^30^ in healthy individuals and patients with mild disease reflected the attenuation of hyperinflammatory and glycolytic pathways. Conversely, transcripts in Q2 and Q3 exhibited divergent patterns between healthy individuals and patients with mild and severe disease (Figure 4D). *SIRPB2*, a gene that modulates cytokine production and innate immunity,^31^ and *IRF3*, a transcription factor crucial for the innate immune response to viral infections,^32^ were reduced in both mild and severe disease patients relative to healthy controls, suggesting blunted antiviral responsiveness in hyperinflammatory monocytes. In contrast, *IFI27*, an interferon-inducible gene associated with antiviral responses,^33^ and *SOCS3*, a suppressor of cytokine signaling elevated during chronic inflammation,^34^ exemplify alleviated immune responses.

Similarly, in NK cells, SwitchClass revealed clear directional transcriptional patterns that disentangle homeostatic regulation from persistent inflammatory and interferon-driven activity (Figures S4A and S4B). Example genes in Q1 and Q4, including *NFATC3*, an inflammation-responsive transcription factor,^35^ *MYO1F*, which is selectively expressed in immune cells,^36^ as well as *GSTP1*, a regulator of oxidative stress,^37^ and *JAK3*, essential for NK cell growth and maturation,^38^ were maintained at comparable levels in healthy controls and mild cases. In contrast, perturbation-aligned genes in Q2 and Q3 included *GIMAP1*, required for NK cell survival and function,^39^ and *SIGLEC7*, an inhibitory immune checkpoint receptor,^40^ as well as interferon-stimulated genes such as *MX1*, a central mediator of antiviral innate immunity,^41^ and *ISG15*, which encodes a ubiquitin-like modifier that restricts viral replication.^42^ Together, these gene expression patterns reveal the decoupling of homeostatic regulatory programs and inflammatory activity in CD14^+^ monocytes and NK cells, characteristic of COVID-19 severity.

## Discussion

Biological systems rarely transition cleanly between conditions such as health and disease. Instead, they often pass through intermediate states that show partial molecular reprogramming. In this study, we introduce SwitchClass, a label-switch classification framework that quantifies the directional molecular behavior in intermediate states and explicitly distinguishes features that align with the baseline state from those that align with the perturbed state. Across controlled simulations and case studies spanning various biotechnologies (proteome, phosphoproteome, and transcriptome modalities) and biological systems, SwitchClass robustly separated baseline-aligned features from perturbation-aligned features for recovering directionally informative molecular profiles. Additional robustness analyses showed that directional rankings were stable across label-noise, feature-correlation, bootstrap, random-seed, downsampling, and quantitative separation analyses. These benchmarks establish that in the presence of intermediate states, explicitly modeling directional alignment provides interpretative advantages over conventional two-group contrast analyses.

Methodologically, SwitchClass differs from prior strategies in two aspects. First, the label-switch design converts the intermediate state problem into two complementary binary classifications that share the same features but invert its grouping. The contrast of feature-importance vectors from these classifiers yields a single directional score, *δ*, that is straightforward to interpret. The two classifiers are best interpreted as complementary discriminative configurations of the same three-state problem, rather than independent statistical estimators. Permutation-based calibration provides an empirical reference for the directional score magnitude, while bootstrap aggregation and repeated random forest fitting reduce sampling and model-level stochasticity. While random forest was employed in the current model, the conceptual framework can be applied with other classifier and feature-importance metrics, allowing practitioners to employ linear models for interpretability or other non-linear models for non-linear effects and to estimate stability via bootstrapping.

From a biological perspective, the quadrant stratification (Q1–Q4) situates each feature within both direction (baseline-aligned vs. perturbation-aligned) and regulation (activation vs. repression), enabling downstream interpretation such as pathway enrichment and network analyses. For example, perturbation-aligned and activated phosphosites can nominate kinase activities that remain pathologically high after dietary reversal, whereas baseline-aligned and repressed transcripts in COVID-19 may indicate restoration of antiviral defenses. Such directed summaries are useful for prioritizing targets for intervention or for monitoring treatment response.

Together, these findings indicate that SwitchClass presents a practical strategy for dissecting directional molecular behavior in systems that exhibit partial alteration. By reframing classification-based feature importance through a label-switch design, SwitchClass quantifies baseline-aligned and perturbation-aligned molecular profiles, using continuous and interpretable scores. The framework enables analyses across the transcriptomic, proteomic, and phosphoproteomic datasets, revealing the mechanisms of adaptation and maladaptation and providing a foundation for comparative and translational studies. It complements, rather than replaces, existing differential and pathway tools. We envisage a typical workflow in which SwitchClass first assigns directional labels to features, followed by enrichment, network inference, and causal hypothesis testing targeted to the relevant quadrants. This modularity should make the method easy to adopt across omic modalities and experimental settings.

### Limitations of the study

There are several limitations that future extensions may address. First, SwitchClass relies on accurate group labels. Performance degraded with increasing label noise, particularly when effect sizes were weak, and the method is, therefore, less suitable for settings with highly uncertain labels or poorly separated phenotypes. Second, feature importance from non-linear models can be affected by correlations among predictors. Our block-correlation simulations and stability analyses indicate that the overall directional recovery remained robust, but individual correlated features can remain partially unidentifiable; conditional-importance or module-level scoring may be more appropriate when the goal is to nominate individual driver features. Third, class imbalance can shift feature-importance estimates. Downsampling analyses showed that directional rankings were stable at the imbalance levels present in our cohorts, up to ∼3.4:1, but more extreme imbalance, such as ≥5:1, and very small sample sizes remain uncharacterized. Fourth, pseudo-bulk aggregation in the single-cell analysis captures donor-level cell type signals while preserving patient-level independence, but it does not resolve within-patient cell-to-cell heterogeneity. Thus, SwitchClass should be interpreted as identifying directional shifts at the donor-level cell-type resolution, rather than modeling continuous cell state variation within individual patients. Finally, the current implementation is designed for three discrete states. Richer time courses, multiple intermediate states, or continuous trajectories would require extensions of the label-switch framework. Although SwitchClass is classifier agnostic in principle, the empirical analyses here used the random forest implementation as default, and alternative model classes may require separate validation.

## Resource availability

### Lead contact

Requests for further information and resources should be directed to and will be fulfilled by the lead contact, Pengyi Yang (pengyi.yang@sydney.edu.au).

### Materials availability

This study did not generate new unique reagents.

### Data and code availability

• No new experimental data were generated in this study. The colorectal cancer plasma proteomics dataset was obtained from Li et al., 2024,^2^ and raw MS/PRM data are available through ProteomeXchange: PXD047207 and iProX: IPX0005221000. The insulin resistance phosphoproteomic dataset was obtained from Fazakerley et al., 2023,^3^ and raw and processed MS data are available through PRIDE: PXD032913. The COVID-19 single-cell transcriptomic dataset was obtained from Schulte-Schrepping et al., 2020,^4^ with single-cell RNA-sequencing (scRNA-seq) data deposited in EGA: EGAS00001004571.
• SwitchClass is freely available from https://github.com/PYangLab/SwitchClass. The original code has been deposited at Zenodo (doi: https://doi.org/10.5281/zenodo.21252910).
• Any additional information required to reanalyze the data reported in this paper is available from the lead contact upon request.

## Acknowledgments

We thank the colleagues from Computational Systems Biology Unit for their support and intellectual engagement. This work was supported by a Robinson Fellowship (G200734) to P.Y.

## Author contributions

Conceptualization, P.Y.; methodology, P.Y. and D.X.; investigation, P.Y. and D.X.; writing – original draft, P.Y. and D.X.; writing – review & editing, P.Y., D.X., and S.L.; funding acquisition, P.Y.; resources, P.Y. and D.X.; supervision, P.Y.

## Declaration of interests

The authors declare no competing interests.

## Declaration of generative AI and AI-assisted technologies in the writing process

During the preparation of this work, the author(s) used a large language model (ChatGPT-5.1 from OpenAI) for copyediting during manuscript preparation. After using this tool or service, the author(s) reviewed and edited the content as needed and take(s) full responsibility for the content of the publication.

## STAR★Methods

### Key resources table

**Table:** 

REAGENT or RESOURCE	SOURCE	IDENTIFIER
**Software and algorithms**		

SwitchClass, v0.99.0	This paper	https://github.com/PYangLab/SwitchClass
SwitchClass original code	This paper	doi: https://doi.org/10.5281/zenodo.21252910
randomForest R package, v4.7-1.2	Breiman^43^	https://cran.r-project.org/package=randomForest
limma R package, v3.66.0	Ritchie et al.^5^	https://bioconductor.org/packages/limma
edgeR R package, v4.8.2	Robinson et al.^6^	https://bioconductor.org/packages/edgeR
edgeR v4 methods	Chen et al.^44^	https://bioconductor.org/packages/edgeR
voom	Law et al.^45^	https://bioconductor.org/packages/limma
RUV normalization	Gagnon-Bartsch and Speed^46^	https://bioconductor.org/packages/PhosR
PhosR R package, v1.20.0	Kim et al.^21^	https://bioconductor.org/packages/PhosR
uwot R package, v0.2.4	McInnes et al.^47^	https://cran.r-project.org/package=uwot
scuttle R package, v1.20.0	McCarthy et al.^48^	https://bioconductor.org/packages/scuttle
SingleCellExperiment R package, v1.32.0	Amezquita et al.^49^	https://bioconductor.org/packages/SingleCellExperiment
PRROC R package, v1.4	Grau et al.^50^	https://cran.r-project.org/package=PRROC
ggplot2 R package, v4.0.3	Wickham^51^	https://cran.r-project.org/package=ggplot2
msigdbr R package, v26.1.0	Subramanian et al.^52^	https://cran.r-project.org/package=msigdbr

### Method details

#### SwitchClass computational framework

SwitchClass was developed to quantify directional molecular behavior across systems comprising three biological states that represent baseline, perturbed, and intermediate states. The method is based on a label-switch strategy that uses complementary binary classifications using models that are capable of ranking feature importance to capture the contribution of molecular features to each direction of change. This design enables systematic detection of features whose intermediate profiles either match to the baseline state or are aligned with the perturbed state.

Specifically, in the current implementation, two random forest classifiers^43^ are trained under inverted label configurations. In the first, termed the baseline-aligned configuration, samples from the baseline and intermediate states are pooled and classified against the perturbed state. In the second, termed the perturbation-aligned configuration, the intermediate and perturbed samples are grouped and classified against the baseline state. These two label assignments are denoted *y*_*B*_ and *y*_*P*_, respectively. Each model yields a feature-importance vector, *Ι*_*B*_ from the baseline-aligned model and *Ι*_*P*_ from the perturbation-aligned model, quantifying the contribution of each feature to class discrimination. The directional importance score for feature *i* is defined as

$$\delta_{i}={Ι}_{B,i}-{Ι}_{P,i}$$

Positive *δ*_*i*_values indicate that a feature contributes more strongly under the baseline-aligned model and therefore exhibits baseline behavior, whereas negative *δ*_*i*_values indicate stronger contribution under the perturbation-aligned model and therefore perturbed behavior. The absolute magnitude |*δ*_*i*_| reflects the strength of directional alignment. The two classifiers are interpreted as complementary discriminative configurations of the same three-state problem rather than as independent statistical estimators. The directional score *δ* is used as a contrast of feature relevance under these two alternative assignments of the intermediate state.

To facilitate interpretation, features are stratified into four quadrants (Q1-Q4) according to the sign of *δ*_*i*_ and the direction of fold-change between baseline and perturbed states. This classification delineates baseline-aligned versus perturbation-aligned features while distinguishing activation and repression patterns within each category. For stability estimation, the training procedure is repeated across 20 bootstrap resamples of samples, and the median *δ*_*i*_ across replicates is reported as the final estimate. Within each SwitchClass run, feature importance is averaged across 25 repeated random-forest fits to reduce model-level stochasticity. The resulting directional importance scores are used to identify molecular subsets underlying the directional organisation of intermediate states.

For statistical calibration of *δ*, sample labels were permuted to construct a pooled signed null distribution of directional scores. For each observed feature, an empirical directional *p*-value was computed from the corresponding tail of the pooled null distribution according to the sign of *δ*_*i*_, and *p*-values were adjusted across features using the Benjamini-Hochberg procedure to obtain FDR-adjusted *q*-values. Permutation calibration used *B* = 100 permutations for simulated datasets and *B* = 500 permutations for the colorectal cancer bulk proteome. Thus, *δ* is retained as a continuous directional ranking measure, while the permutation-derived *p*-values and FDR-adjusted *q*-values provide an additional significance filter for prioritising statistically supported directional features.

To assess the effect of class imbalance on directional rankings, SwitchClass was rerun after stratified downsampling of all groups to the smallest group size. This procedure was repeated across 50 independent downsampling replicates, and the resulting directional scores were compared with scores from the full dataset using Spearman correlation.

#### Simulation benchmark

To evaluate the performance and robustness of SwitchClass, we generated simulated datasets in which the underlying directional behavior of each feature was predefined. Each dataset comprised three groups representing baseline, intermediate, and perturbed states, corresponding to the configuration of the SwitchClass framework. A total of 1,000 features were simulated from multivariate normal distributions with controlled variance *X* ∼ *N*(*μ*_*s*_, 1), partitioned into four behavioral categories. Specifically, 50 features were defined as baseline-aligned with mean pattern (0,*α*, 0) of the three states, 50 as perturbation-aligned with pattern (0,*α*,*α*), 200 as intermediate-specific with pattern (0, 0,*α*), and 700 as null features with no systematic differences (0, 0, 0).

We systematically varied the mean separation between the baseline and perturbed groups (effect size, *α*) and the number of samples per group (*n*) to examine the influence of signal strength and sample size on method performance. Effect sizes were drawn from *α*∈{0.6, 1.0, 1.6}, corresponding to ∼1.5-, ∼2-, and ∼3-fold differences, and sample sizes from *n*∈ {10, 20}, covering a range of realistic experimental settings. For each simulation, we computed directional importance scores (*δ*) using SwitchClass and compared its ability to recover the known feature classes with a conventional pairwise differential expression approach that compares intermediate vs. perturbed and baseline vs. intermediate groups for detecting baseline-aligned and perturbation-aligned features, respectively, using the limma R package.^5^

Performance was evaluated using the area under the precision-recall curve (AUPRC) calculated with the PRROC package.^50^ For each *α* and *n* combination, five independent repetitions (*B* = 5) were generated, and mean ±95% confidence intervals were computed from repetitions. Visualisation of sample-level structure was achieved using UMAP embeddings derived from baseline-aligned, perturbation-aligned, intermediate-specific, or null feature subsets, or full features, demonstrating how directional separation of samples was recovered under different simulation conditions.

Performance was also evaluated using alternative feature-importance ranking methods, including elastic-net logistic regression and gradient boosting. Similarly, cross-validated estimation of (*δ*) was performed using 5-fold cross-validation. Feature-level (*δ*) values were computed per fold and aggregated across folds using the median. This procedure was applied independently to each feature ranking method to assess robustness of directional inference.

To assess robustness to label noise, we randomly reassigned a fraction of sample labels to alternative groups across noise levels of 0%, 5%, 10%, 20%, and 30%. This analysis was performed across the same effect sizes (*α*∈{0.6, 1.0, 1.6}), and performance was evaluated using AUPRC for recovery of baseline-aligned and perturbation-aligned feature classes.

To evaluate continuous partial recovery rather than only endpoint-aligned scenarios, we simulated an additional partial-recovery feature class with mean profile (0,*α*,*βα*), reported in the order baseline, perturbed, and intermediate/recovery. The recovery fraction *β* was swept from 0 to 1 in increments of 0.1, where *β* = 0 corresponds to complete baseline alignment and *β* = 1 corresponds to complete perturbation alignment. The mean directional score of partial-recovery features was then tracked as a function of *β*.

To examine the effect of correlated predictors, we introduced block-wise feature correlation into the simulated datasets. Features were grouped into blocks of size *k* = 5, and within-block correlation was varied across *ρ*∈{0, 0.3, 0.6, 0.9}. Directional recovery was quantified using AUPRC across within-block correlation levels and effect sizes.

#### Experimental datasets preprocessing and downstream analysis

We demonstrate the utility of SwitchClass on three experimentally derived datasets capturing reversible or progressive molecular adaptations in distinct biological contexts. The first dataset is a human plasma proteomic dataset derived from a longitudinal colorectal cancer cohort.^2^ For this dataset, raw protein level intensity values were obtained from the original study and subset to include healthy controls, oxaliplatin-sensitive pre-treatment samples, and post-treatment samples from the stable disease subgroup (SSG). Protein intensities were log2-transformed and filtered to retain those quantified in at least 10% of samples within each group. Missing values were imputed using the PhosR package.^21^ Differential expression analysis was performed using limma, comparing healthy controls to pre-treatment samples. Proteins with an adjusted *p*-value < 0.05 and an absolute fold change of 1.5 were retained as differentially expressed proteins. This curated dataset captures a directional proteomic response across health, disease, and therapeutic stabilization, and was used for all downstream SwitchClass analyses.

The second dataset is generated from a mouse adipose phosphoproteomic experiment profiling dietary perturbation and reversal.^3^ Previously processed phosphosite intensities (log2 LFQ) by PhosR^21^ were obtained from the original study. These values were derived from adipose tissues collected under chow basal conditions, and from chow, HFD, and reversal groups following acute insulin stimulation. To focus on insulin-regulated signaling, we first performed differential analysis within chow animals (insulin vs. basal) and retained phosphosites that were significantly insulin-responsive (Benjamini-Hochberg FDR <0.05) with an absolute fold change of 1.5. The resulting insulin-responsive set was used for all downstream analyses on insulin-stimulated samples. This dataset provides a controlled model for examining phosphorylation events that normalise or remain altered during the reversal of diet-induced insulin resistance.

The third dataset is generated from a single-cell transcriptomic study of peripheral blood mononuclear cells (PBMCs) across COVID-19 disease severity.^4^ Count matrices from the original study were obtained. To ensure sufficient sampling depth per donor and cell type, we first retained only patient-cell type pairs containing at least 20 cells. Pseudo-bulk counts were then constructed by summing gene counts for each patient-cell type combination. We next restricted to cell types with adequate patient representation in each disease group by requiring, for that cell type, at least five patients per group across healthy, mild, and severe. Within each retained cell type, patient-level counts were TMM-normalised and transformed with Voom.^45^ To mitigate unwanted variation while protecting the severity effect, we applied RUV normalisation^46^ on the voom-transformed matrix. Differential expression was then performed between healthy and severe patients using limma to select for the top 1000 genes that vary in expression between the two conditions. For feature-selection sensitivity analysis, this step was repeated using the top 50, 200, 500, and 1,000 differentially expressed genes. Directional scores for shared genes across feature-set sizes were compared using Spearman correlation and sign concordance. Stability was further assessed using 50 stratified patient-level bootstrap replicates in the two strongest cell types, CD14 Mono and NK, and by repeating the analysis across 20 independent random seeds. These steps enable robust modeling of cell-type-specific transcriptional variation at the donor level. Together, these datasets serve as examples of molecular adaptation and incomplete recovery at signaling and transcriptional levels.

For UMAP-based visualisations, group separation was quantified using average silhouette width (ASW). ASW was calculated for each directional feature subset in both the two-dimensional UMAP embedding and the original high-dimensional feature space, and compared with the corresponding all-feature baseline.

### Quantification and statistical analysis

Statistical calibration of SwitchClass directional scores was performed by permuting sample labels to generate a pooled signed null distribution of *δ*. For each feature, an empirical directional *p*-value was calculated from the corresponding tail of this null distribution according to the sign of *δ*_*i*_. *p*-values were adjusted across features using the Benjamini-Hochberg procedure to obtain FDR-adjusted *q*-values. Differential analyses used limma, with significance defined as Benjamini-Hochberg-adjusted *p*-value <0.05 and absolute fold change of 1.5 where indicated.

## Supplemental information

Document S1. Figures S1–S4

Document S2. Article plus supplemental information
