# Supplementary material for: SwitchClass distinguishes baseline- and perturbation-aligned features across intermediate molecular states
Source: Cell Rep Methods. 2026 Aug 12;6(10):101547. doi: 10.1016/j.crmeth.2026.101547 (PMC13721760; doi:10.1016/j.crmeth.2026.101547)
Supplement: Supplementary file 1 [file mmc1.pdf]

Cell Reports Methods, Volume *6*

Supplemental information

SwitchClass distinguishes baseline- and perturbation-aligned features across intermediate molecular states

Di Xiao, Siqu Long, and Pengyi Yang

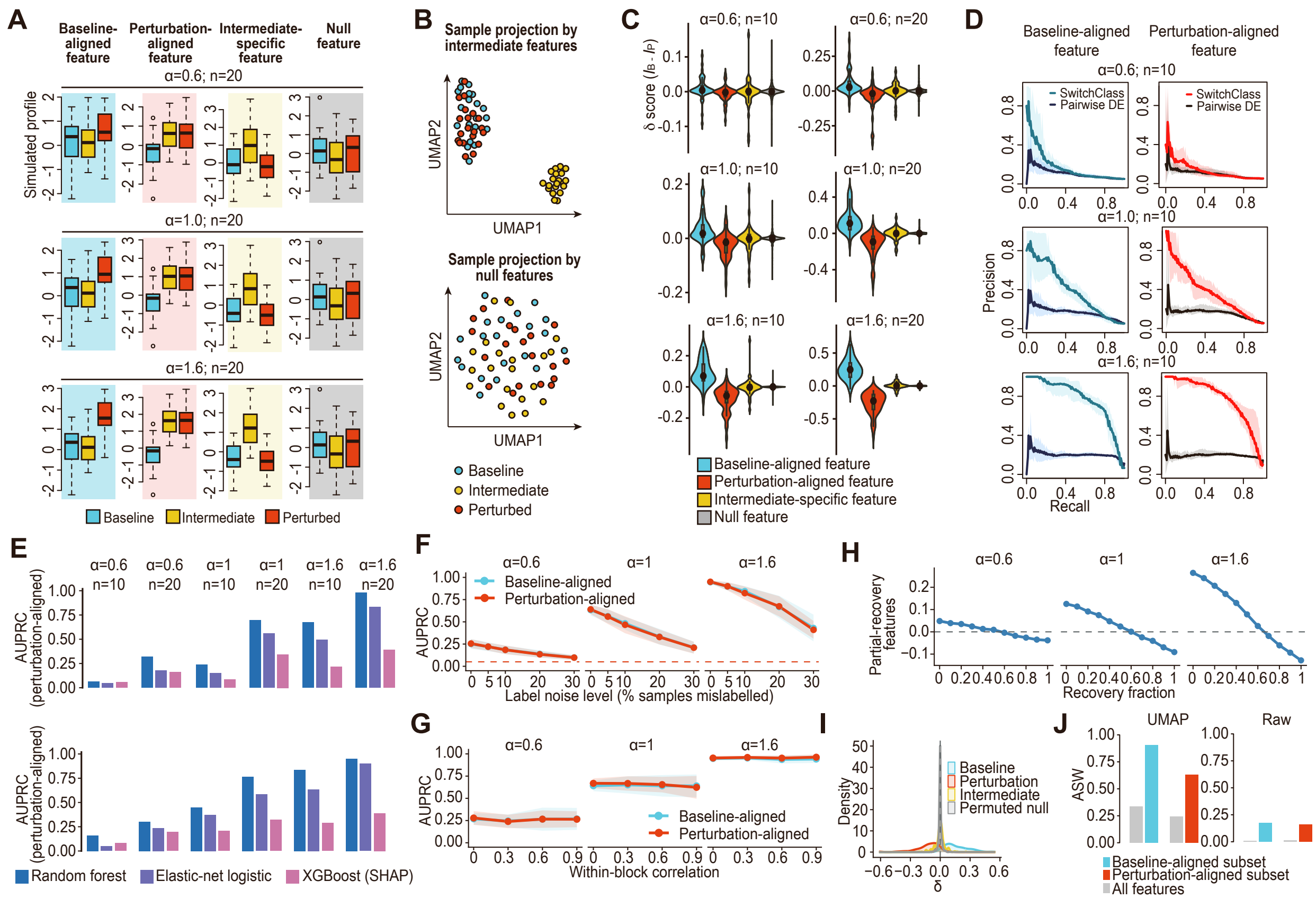

Figure S1. Simulations and benchmarks of SwitchClass under varying effect sizes and sample sizes, related to Figure 1. (A) Examples of simulated expression profiles of baseline-aligned, perturbation-aligned, intermediate-specific, and null features across baseline, intermediate, and perturbed states for three effect sizes ($\alpha = 0.6, 1.0, 1.6$) and sample size of $n = 20$. **(B)** UMAP projections of samples using intermediate-specific and null features. **(C)** Distributions of differential importance scores $\delta = I_B - I_P$ from SwitchClass across feature classes, effect sizes ($\alpha = 0.6, 1.0, 1.6$), and sample sizes ($n = 10, 20$). **(D)** Precision-recall performance of SwitchClass in detecting baseline-aligned and perturbation-aligned features compared with pairwise differential expression (DE) across varying effect sizes ($\alpha = 0.6, 1.0, 1.6$) and a sample size of $n = 10$ for each state. Shaded areas denote 95% confidence intervals derived from bootstrap resampling across simulation replicates. **(E)** SwitchClass performance with different feature-ranking approaches. AUPRC for identifying baseline-aligned (left) and perturbation-aligned (right) features using SwitchClass with random forest, elastic-net logistic regression, and gradient boosting with SHAP values. Results are shown across simulated datasets with varying signal strength (α) and sample size (n). **(F)** Robustness to random label noise. AUPRC for recovery of baseline-aligned and perturbation-aligned features across label-noise levels from 0% to 30%, effect sizes ($\alpha = 0.6, 1.0, 1.6$), and a sample size of $n = 20$. Shaded areas denote standard deviation across simulation replicates. **(G)** Robustness to block-wise feature correlation. AUPRC for recovery of baseline-aligned and perturbation-aligned features across within-block correlation levels ($\rho \in \{0, 0.3, 0.6, 0.9\}$; block size $k = 5$), effect sizes ($\alpha = 0.6, 1.0, 1.6$), and a sample size of $n = 20$. Shaded areas denote standard deviation across simulation replicates. **(H)** Mean directional score δ of partial-recovery features as a function of recovery fraction β, where $\beta = 0$ corresponds to a baseline-like intermediate state and $\beta = 1$ corresponds to a perturbation-like intermediate state, across effect sizes ($\alpha = 0.6, 1.0, 1.6$) and a sample size of $n = 20$. **(I)** Permutation-based significance calibration. Density of the pooled permutation null distribution of δ and the observed δ distribution stratified by true feature class, shown for $\alpha = 1.0$ and $n = 20$. **(J)** Average silhouette width (ASW) for baseline-aligned, perturbation-aligned, and all-feature subsets in the two-dimensional UMAP embedding and the original high-dimensional feature space.

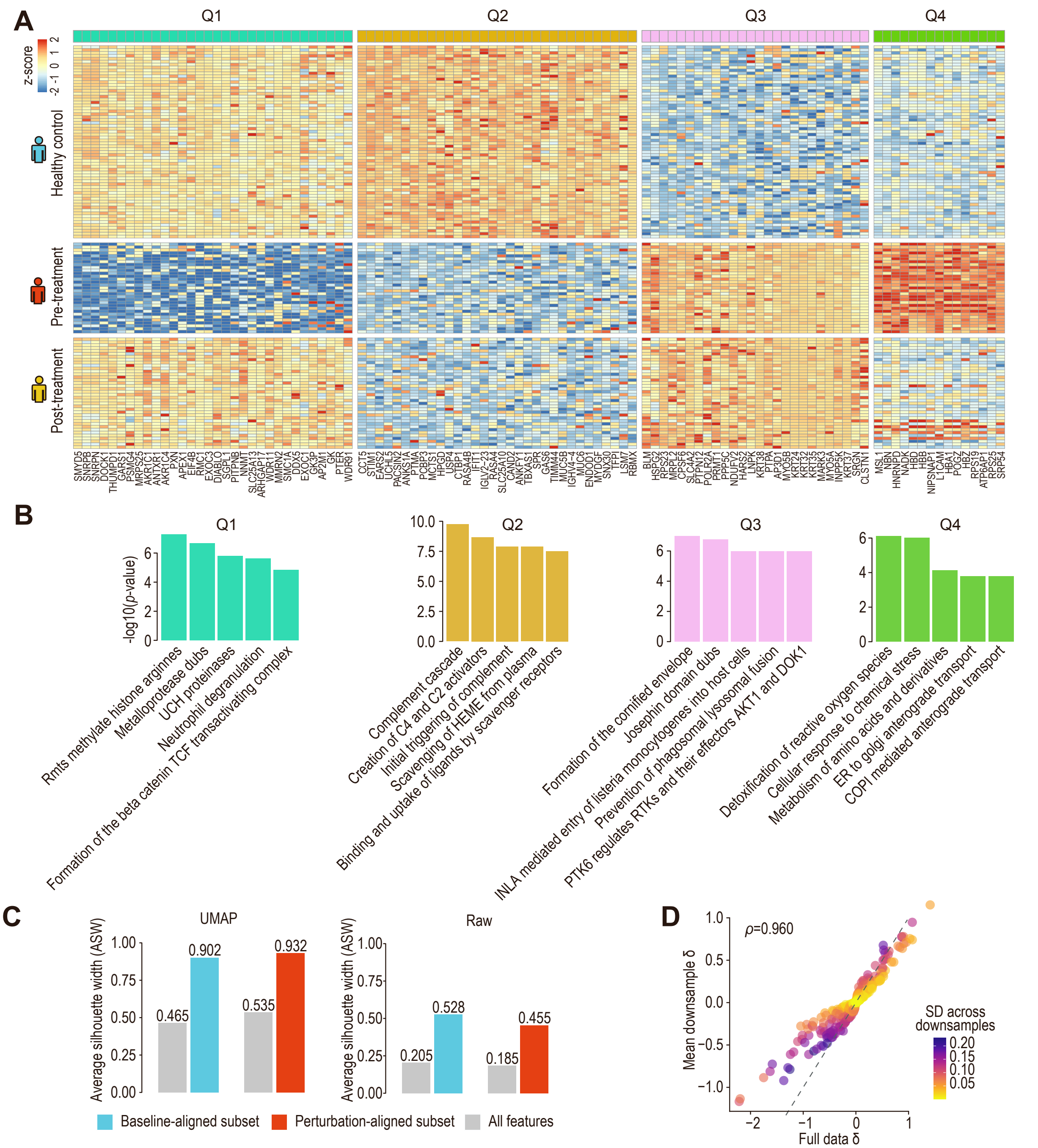

Figure S2. Plasma proteomic expression and SwitchClass identified baseline-aligned or perturbation-aligned proteins in colorectal cancer and after its treatment, related to Figure 2. (A) Heatmap of proteins identified by SwitchClass as baseline-aligned (Q1, Q4) or perturbation-aligned (Q2, Q3) across healthy controls, and in colorectal cancer pre-treatment and post-treatment samples. **(B)** Pathway enrichment analysis of quadrant-specific proteins using the Reactome database. **(C)** Average silhouette width (ASW) for baseline-aligned, perturbation-aligned, and all-protein subsets in the two-dimensional UMAP embedding and the original high-dimensional proteomic space. **(D)** Directional scores from the full colorectal cancer proteomic dataset compared with mean directional scores after stratified downsampling to the smallest group size across 50 independent replicates. Points are coloured by the standard deviation across downsampling replicates.

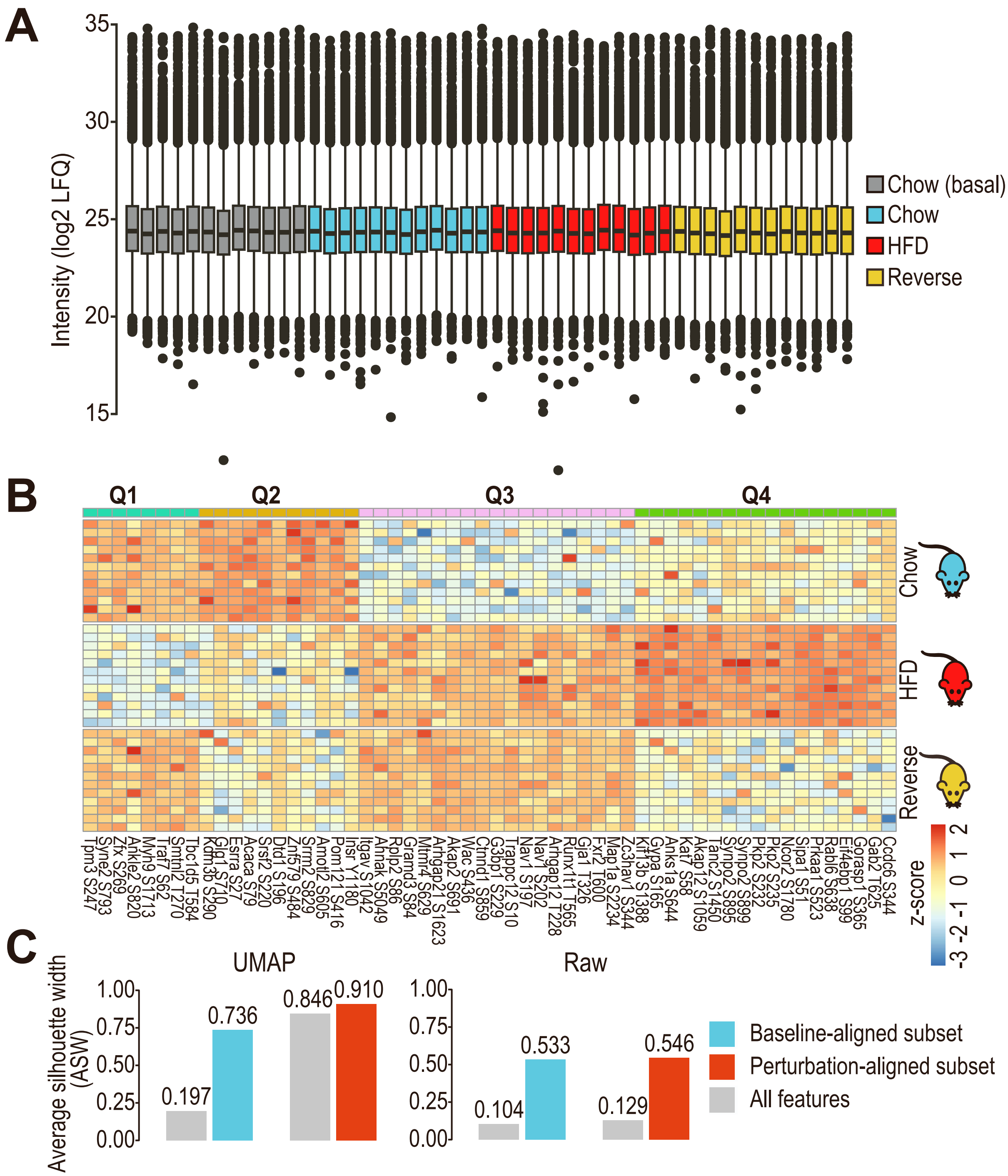

Figure S3. Phosphoproteomic data distribution and SwitchClass identified baseline-aligned or perturbation-aligned phosphosites across dietary perturbation and reversal, related to Figure 3. (A) Boxplots showing the distribution of log2 LFQ intensities across all samples, including basal chow and insulin-stimulated chow, high-fat diet (HFD), and reverse groups. **(B)** Heatmap of phosphosites identified by SwitchClass as baseline-aligned (Q1, Q4) and perturbation-aligned (Q2, Q3) in mice from chow, HFD, and reverse states. **(C)** Average silhouette width (ASW) for baseline-aligned, perturbation-aligned, and all-phosphosite subsets in the two-dimensional UMAP embedding and the original high-dimensional phosphoproteomic space.

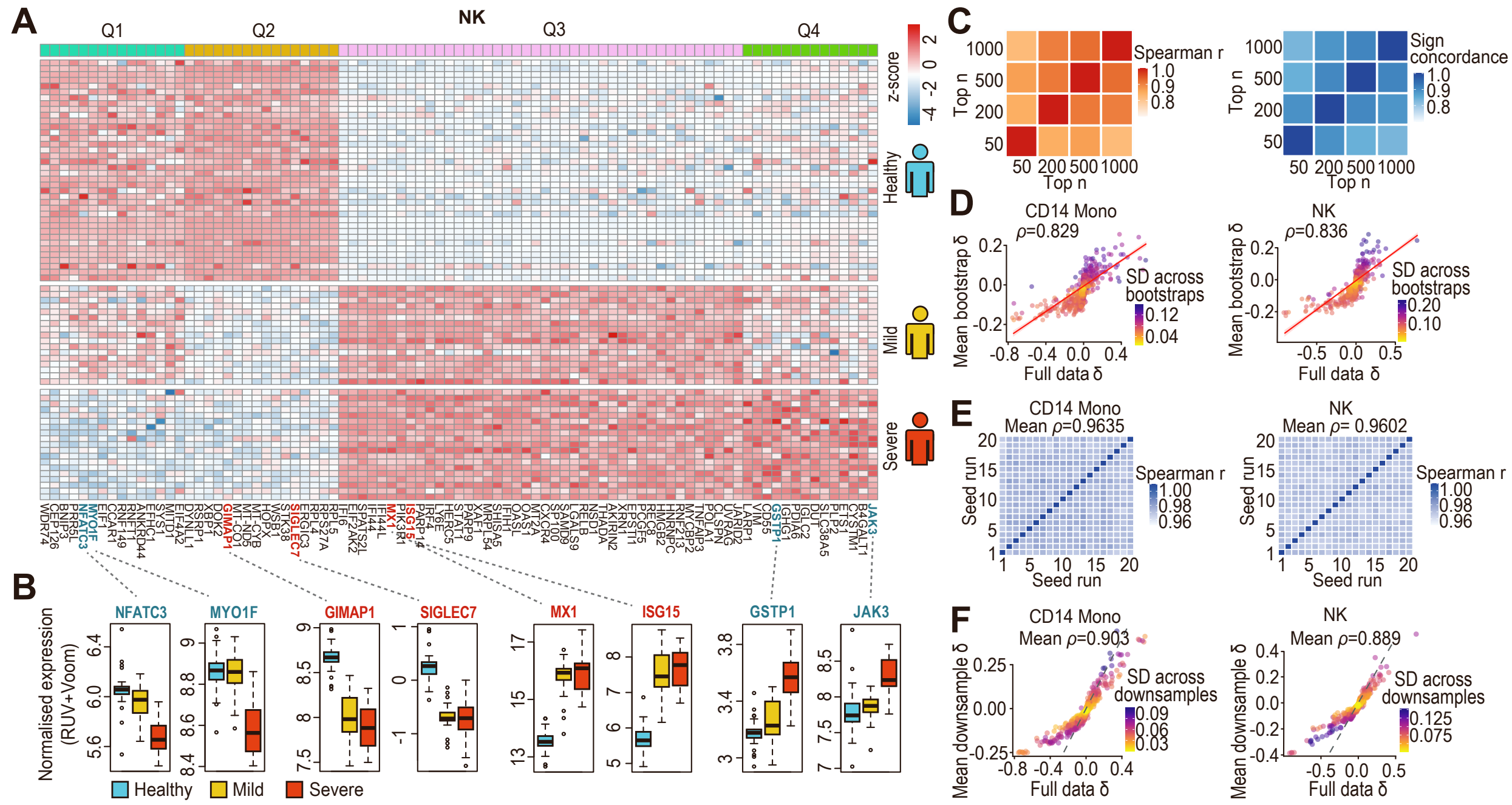

Figure S4. SwitchClass identified baseline-aligned or perturbation-aligned genes in NK cells in healthy individuals, and mild and severe COVID-19 patients, related to Figure 4. (A) Heatmap of expression profiles of genes identified by SwitchClass across the four quadrants in NK cells. **(B)** Representative baseline-aligned (Q1, Q4) and perturbation-aligned (Q2, Q3) genes in NK cells in healthy individuals and mild and severe COVID-19 patients. **(C)** Sensitivity of directional scores to the number of genes retained for SwitchClass analysis, shown by Spearman correlation and sign concordance across top-gene thresholds. **(D)** Directional scores from the full single-cell analysis compared with mean directional scores across bootstrap replicates for $CD14^+$ monocytes and NK cells. Points are coloured by the standard deviation across bootstrap replicates. **(E)** Pairwise Spearman correlations of directional scores across 20 independent random-seed runs for $CD14^+$ monocytes and NK cells. **(F)** Directional scores from the full single-cell analysis compared with mean directional scores after stratified downsampling for $CD14^+$ monocytes and NK cells. Points are coloured by the standard deviation across downsampling replicates.
